# Global Dental Publications in PubMed Databases between 2009 and 2019—A Bibliometric Analysis

**DOI:** 10.3390/molecules25204747

**Published:** 2020-10-16

**Authors:** Faris Yahya Asiri, Estie Kruger, Marc Tennant

**Affiliations:** 1 Department of Preventive Dentistry, College of Dentistry, King Faisal University, Al-Ahsa 31982, Saudi Arabia; 2 International Research Collaboration—Oral Health and Equity, School of Human Sciences, Faculty of Science, The University of Western Australia, Perth WA6009, Australia; estie.kruger@uwa.edu.au (E.K.); marc@ircohe.net (M.T.)

**Keywords:** dentistry, bibliometric, citations, h-indexes, publications, global

## Abstract

The objective of this study was to evaluate the publications in the field of dentistry on the PubMed database over a span of 10 years, from 2009 to 2019. Articles published between January 2009 to December 2019 were searched for in the MEDLINE database via PubMed. Data analysis was done using *R*-base packages, including the specialized *R*-packages Bibliometrix and String. For descriptive statistics and sequence charting, SPSS version 23.0 was used. A total of 104,975 articles were extracted, with a total of 153,530 authors in the given time frame. The proportion of articles steadily increased from 2009, plateauing at its peak from 2010 to 2016, and then seeing a decline from 2017 to 2019. Journal articles (60.58%), comparative studies (16.05%) and case reports (10.8%) were recorded as the most reported type of publication globally, accounting for 81.43% of the total documents extracted. All the articles came from 81 countries, with the USA reporting the greatest number of published articles (45,911). Dentistry proves to be a multi-faceted arena and many researchers and authors around the globe are contributing to the burgeoning literature over time.

## 1. Introduction

Bibliometrics is a type of analysis (quantitative and/or qualitative), implemented for studying academic literature which have some common attributes (e.g., study topics), and it describes temporal progress in different aspects of literature including the subject field, trends in publication, productivity of authors, utility of research and sometimes helps in forecasting future patterns of the literature [1,2]. Bibliometrics can be termed as the science of measuring research success, and it has evolved over time [3], mainly due to the establishment of eminent research websites and networking processes like Research Gate and Google scholar [4]. These research sites offer a huge platform containing organized information, through which professionals and their networks can explore different topics to define and harness their performance metrics, such as research profiles or scores, direct invitations to publish in journals, citation counts, h-indexes, g-indexes and i10-indexes [4]. Many specialized journals in dentistry also reported many bibliometric types of papers [5].

Data publication occurs because of researchers’ desire to place the information in the public domain as a reference, and in order to provide relevant data sources for analysis on any aspect of a specific topic [6], such as dental health. Research in the field of dentistry is particularly numerous because this area is broad and includes a range of topics [1]. New subjects unfold daily, and dental researchers take on the role of developing precise reports in the various studies that they conduct.

There is enough evidence on the use of bibliometric methods in the field of medical science and health [4,7,8], but it is limited in dentistry to only a few topics—prosthodontics [9], orthodontics [10,11], oro-facial pain [12,13,14], implantology [15,16] and general dental health [9,17]. Some of the bibliometric studies related to dentistry deliberated on specific countries or regions [18,19,20] and others focused on specific databases like International Scientific Indexing (ISI), Scopus or PubMed [3,21]. Historically, dental publications span over a long period of time and the publication of new data sources is always in progress across the world, as evidenced in the consistent publication of journals internationally. In the current discussion, the focus shall be only on those featured in PubMed between 2009 and 2019, as in this era such publications not only encompassed new concepts in the field, but also reflected on past events and ideas (before 2009), presenting a broad perspective on dental health.

In this study, PubMed is the only database used which hosts numerous research journals, providing access to publications on a variety of study topics. The search process is flexible [3], offering users a range of opportunities through which one can modify his/her search criteria, e.g., date search option is customizable, thus appropriate for analyzing numerous publications based on the desired time period. The PubMed database allows for the specification of topic under investigation, such as oral health, and the resulting publications will only concern that topic. In this regard, users can focus on different sub-disciplines within the main field; thus, among the list of search results, users can access many interrelated topics under the broader subject. Additionally, users can also access information in bits, as the database gives options to view only abstracts of articles, reviews of articles or the full texts of the publications. Moreover, categories of journals can be selected, such as those on dental or nursing, and the data sources can be classified according to relevance, best match, first author, last author, title, journal and publication date; recently, sorting options have also been added.

## 2. Results

The search criteria yielded a total of 104,975 articles, published by a total of 153,530 authors. There were 7758 authors of single-authored articles, publishing a total of 15,209 articles, and 145,772 authors of multi-authored articles. On average, each article was co-authored by approximately four authors. Including duplicates, there were a total of 441,286 author appearances across the articles. English was the primary language of publication by an overwhelming margin, 93.6% (98,290), with Chinese the second most common publication language, 2.6% (2789), followed by French, >1% (944), Russian, >1% (810), and Dutch, >1% (571), respectively.

The largest number of articles were published in 2014, making up 10.5% of all articles retrieved, closely followed by 2013 and 2011 with 10.5% and 10.4% of all articles, respectively (Table 1). The lowest number of articles, 3207 (3.1%), were published in 2019. Of the 104,975 articles published over the study period, 71.3% were published between 2010 and 2016.

Table 2 represents the statistics on Global Dental Publications from 2009 to 2019. Positive values for mean Average Change Rate (ACR) depict the net percentage increase, while the negative values reflect the net depreciation rate for the whole study period. Only two types of publications showed a net increase over time, i.e., Journal article, 4.17%, and Letters, 11.47%, respectively, whereas the rest of the publication types decreased as a whole during the study period, with comparative studies showing a maximum depreciation percentage (−17.56%) over the time (Table 2). Controlled clinical trials and historical articles also decreased at a high rate, i.e., −15.69% and −15.97%, respectively. Journal articles (60.58%) were the most common type of document, followed by comparative studies (16.05%) and case reports (10.8%). Together, journals, comparative studies and case reports accounted for 81.43% of the documents. Also, common but less frequent were editorials (2654), letters (2393), evaluation studies (1794) and clinical trials (1114) (Table 2).

**Figure 1:**
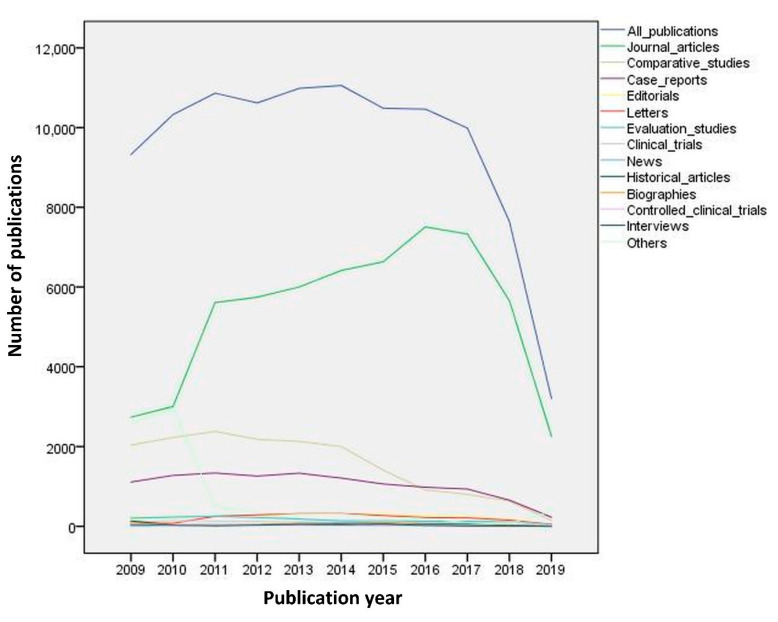
Sequence chart, Global Dental Publications and impact factor characteristics of most relevant sources with Journal Citation Reports (JCR) 2009 to 2019. Note: Non-JCR most relevant sources are not included.

The numbers of each publication type from 2009 to 2019 are shown in the Figure 1. Non-JCR journals were not included in this figure. From the start of study to year 2017, “journal articles” followed an upward trend, “comparative studies” gradually decreased, while the rest of the publication types remained steady. A sharp decline thereafter in “all publications” and “journal articles” is also evident. The impact factors of most relevant sources having Journal Citation Report (JCR) are shown in Table 3, while Table 4 shows the most relevant sources which were assigned no impact factors. Both these tables show the top sources of articles on dentistry from 2009 to 2019. The American Journal of Orthodontics and Dentofacial Orthopedics is the number one source with 2707 articles and the only one to have published more than 2500 articles. The next journal on the list, with 2497 articles, is the British Dental Journal. The Journal of Oral and Maxillofacial Surgery and the Journal of Endodontics round out the top four sources with more than 2000 articles each. Of the top 20 sources, the top 18 journals published over 1000 articles each.

**Figure 2:**
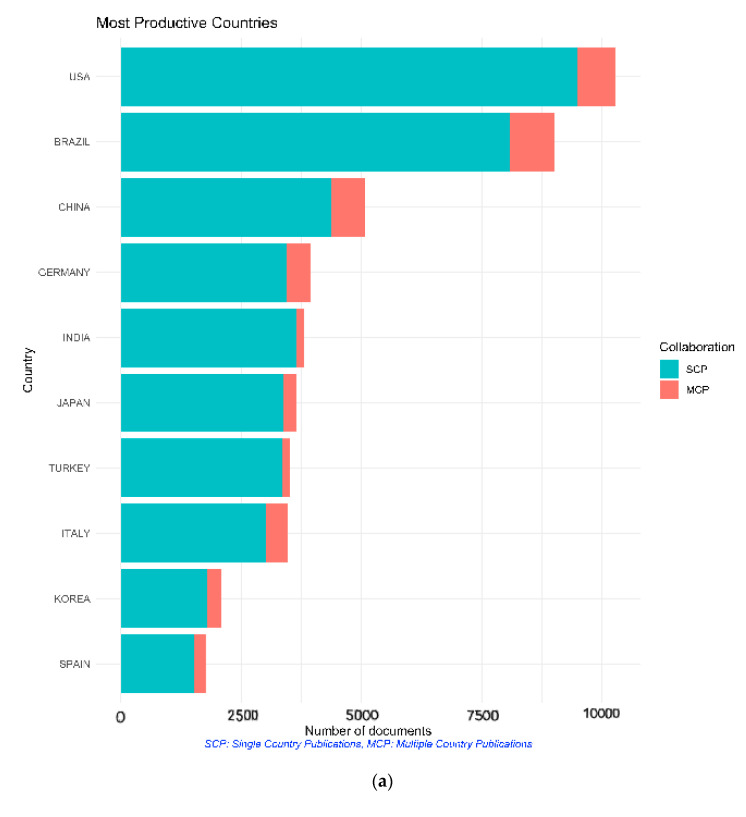

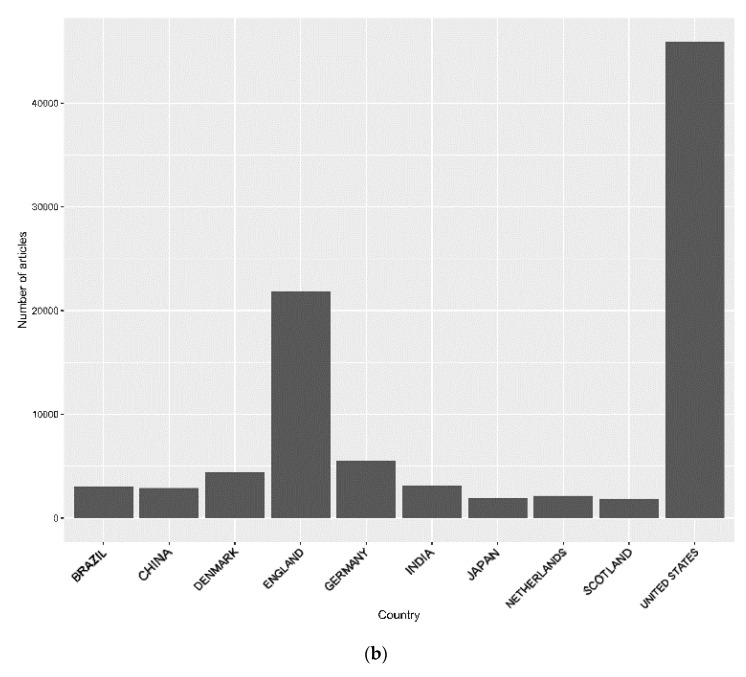
Most productive countries with respect to number of dental publications, 2009 to 2019. (**a**) Based on the count of the corresponding authors from each country for both single country publications (SCP) and multiple country publications (MCP). (**b**) Based on the count of articles published for each country.

Several analyses were carried out to examine the geographical distribution of the publications. The countries of corresponding authors and publications were analyzed, and the number of citations per country were counted. Figure 2 shows the countries claiming the most corresponding authors, across both single- and multiple-country publications. The largest number of corresponding authors came from USA, with 10,281 articles, while the Brazilian authors came in second, publishing 9021 articles. Each of the top eight countries can claim over 3000 articles published. North and South America, Europe and Asia are all represented in this top ten list.

The retrieved articles were published in 81 countries. The USA had the largest number of published articles (45,911), followed by England (21,835) and Germany (5564). The countries to publish the least articles were Indonesia, Luxembourg, Mali, Papua New Guinea, the Republic of Korea and Zimbabwe—each had a single article published during the study period. Fourteen countries had more than 1000 articles published during the study period. Once again, the USA was the most productive country based on both single country publications and multiple country publications, followed by Brazil. The retrieved articles covered topics ranging from human studies, chemistry, surgery, pathology, therapy, physiology, epidemiology to etiology, and were cited in other related works. Articles published in the USA received by far the most citations, with 36,082, superseding Brazil with 23,029 total citations.

The USA topped the list of total number of publications, single as well as multiple author publications and the list of citations both total and per article average (Table 5). 

The h-indices for the most productive authors were calculated and are shown in Table 6 alongside the total number of citations of their work. The h-index is a metric that measures both the productivity and citation impact of an author (h-indexes are reported as they were at the time this study was carried out). Lang, N.P and Zhang, Y have the highest h-indices in the dataset, both having an h-index greater than 16, implying they both published at least 16 articles with at least 16 citations each. Out of the ten most productive authors, Li, J, had the most citations, i.e., in 1729 (Table 7).

The number of articles published by each author were also counted and an analysis of their quality conducted. The most productive author was Wang, Y, who published 394 articles, followed by Zhang, Y with 305 articles. Nine of the most productive authors published over 200 articles each. The top ten most active authors published a total of 3766 articles over the research period.

**Figure 3:**
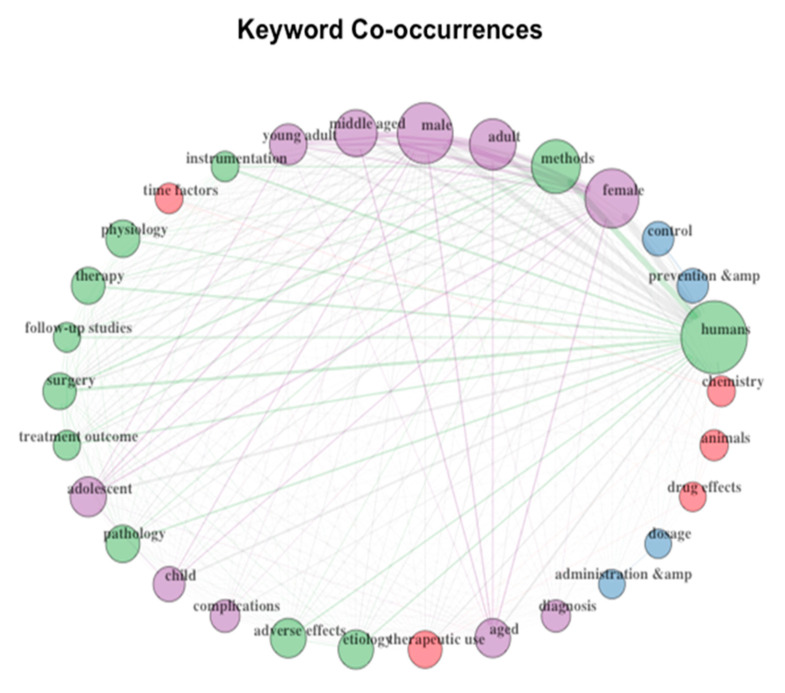
Keyword co-occurrences.

The keywords occurring most frequently were visualized and presented in a network visualization map. The top ten most frequent keywords include “humans”, “methods”, “female”, “adult” and “middle aged”. The frequent keywords are grouped into four clusters. The first cluster includes keywords pertaining to physical health such as “human”, “methods” and “instrumentation”. The second cluster includes keywords representing ages and diagnoses. The third cluster is made up of keywords like “time factors”, “chemistry” and “drug effects”. The last cluster is for keywords pertaining to mental health and laboratory examination of samples: example keywords for this fourth group include “methods”, “physiology”, “treatment outcome” and “adverse effects”. Figure 3 is a graphical visualization of the keyword co-occurrences across the four clusters.

## 3. Discussion

The traditional method of bibliometric analysis and quantified descriptions of total scientific publications was extended in this study. The study also applied visualization techniques, with no restrictions on the language used or document type to comprehensively analyze the patterns of publication in the realm of dentistry from 2009 to 2019. Total articles emerged in excess of one million after the abovementioned search criteria were implemented. The analysis showed a steady incline in the publication trends from 2009 (8.9%, 9325 articles) peaking in 2014 (10.5%, 11,059 articles). A noteworthy increase in the number of scientific dental literature was noticed in this period, mostly journal articles (60.58%, 63,600), comparative studies (16.05%, 16,858) and case reports (10.8%, 11,334). After that, the number of publications decreased each year to a low of 3207 (3.1%) articles for 2019.

Commonly reported types of documented literature were journal articles, comparative studies and case reports, while the less common were evaluation studies, clinical trials and controlled clinical trials. This implies that focus of the dental fraternity globally was on the descriptive side of dentistry more than the analytical during the period analyzed, fixing on the generation of hypothesis rather than testing it, including reports on unusual findings within dentistry, as indicated by the fact that case reports was also amongst the top three document types. It also indicates a possible amount of decreasing facilitation in controlled clinical trials, waning interest in the specific genre of clinical trials and evaluation studies, declining funding both public and private [22,23]. The top three most commonly reported types of document in the literature also indicate that original research was the focus amongst the dental fraternity worldwide, as opposed to reviews [24]. The relatively low numbers of systematic reviews, meta-analyses and randomized controlled trials might be of concern, as these provide the highest level of evidence according to the hierarchy of research evidence [25]. It is known however, that randomized controlled trials in dentistry are seen as very expensive, very slow, difficult to recruit suitable patients, and results are unhelpful when inconclusive [26]. Journal articles and comparative studies, the top two findings, also indicate that an environment conducive to innovative research was encouraged worldwide, particularly with most of the work hailing from the USA, Brazil, China and Germany [27].

Although the article language was not restricted to just English in our search strategy, it turned out to be the primary language of publication (93.6%, 98,290). Language did not show a variety throughout the study period, culminating with English as the primary mode of communication in literature worldwide by a staggering margin as compared to the rest. This is also in consideration of the fact that English remains the first language for communication worldwide, both spoken and written, as opposed to languages like French and Dutch, which collectively accounted for less than 1%.

The geographical distribution of scientific productivity in our field of interest hail mostly from the USA, with 10,281 articles, across 9483 single-country publications and 798 multiple-country publications. This finding is consistent with many other bibliometric analyses conducted in relation to dentistry-related topics, including Ahmed et al. [28], Liu et al. [29] and Lee et al. [22], to name a few. Any specific geographical distribution of scientific literature reflects the research culture, differing capabilities and technological expertise about that particular area under question. Differing research capabilities also rely on the available population for research and the socio-economic demographics of the specific geographical location in focus. Also, our search strategy included extracting articles from MEDLINE using only the PubMed database, which does not encompass all biomedical journals.

In our case, the USA, as expected, claimed the most corresponding authors, in both single- and multiple-country publications, followed by Brazil (9021), especially for corresponding authors in multiple-country publications (935), and China (5065), which, in comparison to the USA, are both developing countries containing limited resources, an identifiable research population available for studies and research prowess. Surprisingly though, ranking five in the top countries’ list was India (3808 articles), with 3654 single-country publications and 154 multiple-country publications, showing increased interest in academic research within dentistry in the subcontinent, but with an inevitable lacking of collaboration with researchers from other countries.

The research productivity attributed mainly to the northern and western side of the world. This also counts for the fact that significant sources of funding, recruitment, promotion and tenure in academia are mostly concentrated in this part of the world, creating a proactive and cooperative environment for research creativity and productivity [27]. The least productive countries, hence, were of the order of Indonesia, Luxembourg, Mali, Papua New Guinea, the Republic of Korea and Taiwan, contributing a single article each to the total literature. Correspondence in the modern era with speedy internet services and the fast emerging concept of global village making everything just a click of the button away, hence facilitates researchers and potential authors worldwide to increasingly collaborate on research articles, also accounting for quick searches online and a sophisticated, pinpoint approach to databases to retrieve articles for analytical studies, such as this one [30].

Variations in the names of the authors, possibly hailing as Asian in terms of origin, could be due to the fact that authors sometimes are students from a developing country who may be residing for a short while in a developed country for research or training purposes. Likewise, one corresponding author may present affiliations of different countries in different publications, but the overall impact is not substantial due to limited number of such authors [29].

The h-index of an author is used to evaluate the scientific production by that particular author in terms of publications, measuring the overall impact of the work done by the researcher. It is calculated by sorting the published articles of the said author in a citation index in descending order by the number of citations of the articles. Then, the articles are counted in the list from top to bottom, and when the number of the article rises above the count of citation of that particular publication, then the amount of the preceding publication is considered as the h-index of that specific author [31]. Lang, N.P and Zhang, Y had the highest h-indices (17 and 16, respectively) at the time of the analysis, but their productivity in terms of publication number was not the highest, fluctuating between twenty and forty during the study period. This infers that the number of publications may not always determine the quality of these documents in terms of their significance whether the specific publication has influenced another research or not [32].

As total scientific output in the field of dentistry was analyzed during the study period of 2009–2019, the top-most relevant sources were included in our study too. Among the top four were *American Journal of Orthodontics and Dentofacial Orthopedics* (2707 articles, average Impact Factor 2009 to 2019 (aIF): 1.671), the only journal which had published more than 2500 articles, in first place, *British Dental Journal* (2497 articles, aIF: 0.914) in second place, *Journal of Oral and Maxillofacial Surgery* (2222 articles, aIF: 1.775) in third place, and *Journal of Endodontics* (2093 articles, aIF: 3.626) in fourth place. These journals have particular dental focus, are content-specifi, and have high impact factors collectively, indicating them to be esteemed within the dental literature, making it important to include focused journals as part of the bibliometric analysis, adding rigor and validation to the results [22,24].

The *American Journal of Orthodontics and Dentofacial Orthopedics* has been publishing scientific literature for more than 100 years now, competing as a leading resource for orthodontic and related research, including all aspects of orthodontic treatment, confining the journal to the specialty of orthodontics under the umbrella of dental discipline. The *British Dental Journal* is an international peer-reviewed journal encompassing clinical, basic and scientific aspects of dentistry, i.e., a wide range of topics being covered through publications in the journal. The *Journal of Oral and Maxillofacial Surgery* comprehensively focuses on significant developments within the discipline of oral and maxillofacial surgery, with innovative advancements in temporomandibular disorders, dentoalveolar and facial trauma and surgeries, head and neck cancer and treatment modalities, also pinpointing diagnosis and ongoing influx of new medicines and therapies. The *Journal of Endodontics* stands 13th out of the 91 top journals around the world. It focuses on scientific publications and advancements in the world of endodontics: pulp conservation, regeneration, endodontic treatments and innovative instrumentation techniques and armamentarium, again confining this journal to a particular specialty of endodontics within the field of dentistry. These journals significantly stood out on the top of the list for the most relevant sources of publications for the study period defined, being consistently considered necessary within the specified field of dentistry and associated sub-specialties. A limitation is that even though these journals were high on the impact factor arena evenly over the years, the quality of scientific work is not distinguishable based on just that as the citation does not specify the quality of work being produced.

Though, an exciting finding at the bottom of the list of relevant resources was the *Journal of Dental Education* (aIF: 1.07). This specific journal is published by the American Dental Education Association, focusing on educational research and innovative teaching methodologies, systematic reviews and meta-analysis of clinical research within the dental domain; all-in-all, a highly scientific journal proving to be top-most in the academic aspect of dentistry. Thus, the fact that this journal was at the bottom of the list with total publications amounting to 964 between 2009 and 2019 is representative of the existence of negligence within the field of dental education of the dental fraternity worldwide. Also, pointing to the fact that worldwide, the views are still focused on curative and rehabilitative phases of dentistry and allied dental specialties as opposed to the preventive aspect [33].

The most cited scientific publications are discussed in Table 6, it showed that the top-most article with the highest total citation count of 475 has discussed clinical guidelines channeling management and long-term care of obstructive sleep apnea in adults with particular emphasis on providing instructions to general surgeons and dentists [34]. The second most cited published manuscript with a total citation count of 359 addressed the prevalence of periodontitis among adults in the USA, including extent of the disease and severity [35]. The manuscripts in third and fourth place had total citation counts of 250 and 246, respectively [36,37]. The rest of the publications consistently had a total citation count between 186 and 198. Citations are indicative of major advancements or innovations within the specified field of interest [32]. Also, to pay homage to the scientific contributions by fellow researchers around the globe. However, the phenomenon of self-citation still exists, even if negligent in high volume of figures [32]. It is also important to acknowledge that number of citations per article is affected by year of publication, and older publications would attract more citations than recent ones [38]. It has been argued that the true impact of an article cannot be determined for at least two decades after its publication [38]. This phenomenon can also be explained by a disposition to follow a pattern in scientific society, leading to a “snowball effect” of citations, because authors are inclined towards a publication that is already abundantly cited, rather than assessing its relevance and quality [38].

The most relevant keywords discussed frequently across the articles included “humans”, “methods”, “female”, “adult” and “middle-aged”. Keyword co-occurrences have also been visualized in a concept map (Figure 3) as they emerged in clusters divided into four groups. The first cluster related to physical health across the literature, the second cluster includes keywords for ages and diagnosis-related, and the third and last included words “time factors”, “chemistry”, “drug effects”, “physiology”, “treatment outcome” and “adverse effects”. Co-occurrences of words tend to misinterpret the ‘syntactical context’ being used of the phrase concerning the specific publications they occur in. The representation of keyword co-occurrences through word maps and visually presenting them holds no real meaning as there appears to be no systematic way of interpreting them as such. On the contrary, an in-depth analysis of such words is recommended as opposed to co-occurrences [32].

Limitations of every study are imminent and vital to discuss. Scientific output even though measured by the amount of literature being published yearly is a clear indicator of productivity amongst research domains, nevertheless, other sources should also be considered, like books, conferences and seminars, patents and patent citations. Our search database was primarily MEDLINE through PubMed, which even though it is the most widely used database, it still does not contain all biomedical journals in the vast literature domain. Some other limitations were the number of citations an article receives does not necessarily indicate its quality, and the data did not differentiate early career researchers from experienced researchers, so comparisons were made between researchers at different career stages.

## 4. Materials and Methods

This analysis quantifies and describes the production of scientific articles on dentistry for the period 2009 to 2019. Data was collected from the MEDLINE database and searched via PubMed, an interface to the MEDLINE data. The data was queried, organized, analyzed and visualized using R, a programming language for conducting statistical analyses and creating accompanying graphics. This analysis used R-base packages and the specialized R packages bibliometrix [39] and stringr [40].

A search was conducted for articles published between January 2009 and December 2019 that included the terms “Dentistry”, “Evidence-Based Dentistry”, “Public Health Dentistry”, “Pediatric Dentistry”, “Geriatric Dentistry”, “Forensic Dentistry”, “Dentistry Operative”, “Community Dentistry”, “Dental Care for Disabled”, “Prosthodontics”, “Oral Health”, “Oral Medicine”, “Endodontics”, “Orthodontic”, “Pathology Oral”, “Periodontics”, “Surgery, Oral” and “Diagnosis, Oral”. The exact search criteria used was ‘(((((“Dentistry”[Mesh] OR “Evidence-Based Dentistry”[Mesh] OR “Public Health Dentistry”[Mesh] OR “Pediatric Dentistry”[Mesh] OR “Geriatric Dentistry”[Mesh] OR “Forensic Dentistry”[Mesh] OR “Dentistry, Operative”[Mesh] OR “Community Dentistry”[Mesh] OR “Dental Care for Disabled”[Mesh] OR “Prosthodontics”[Mesh]) OR “Oral Health”[Mesh]) OR “Oral Medicine”[Mesh]) OR “Endodontics”[Mesh]) OR “Orthodontics”[Mesh]) OR “Pathology, Oral”[Mesh] OR “Periodontics”[Mesh] OR “Surgery, Oral”[Mesh]) OR “Diagnosis, Oral”[Mesh] AND (“2009/01/01”[PDAT]: “2019/12/31”[PDAT])’. No language or document type restrictions were applied to the search since the aim was to analyze patterns for all publications in this field.

The analysis of variance (ANOVA) was implemented in GraphPad Prism, while the descriptive statistics and sequence chart were done in SPSS 23.0. Descriptive statistics were shown as mean values and standard deviation with “all publications” showing maximum mean and standard deviation, followed by “journal articles” and then the rest. Average Change Rate for every two consecutive years, calculated as (current year total—previous year total)/previous year total) were implemented and their cumulative mean was presented in percentage.

## 5. Conclusions

A total of 104,975 articles were published on dentistry and related fields between 2009 and 2019 and indexed by MEDLINE. The number of articles published each year virtually persisted from 2009 to 2017 but then started declining, to a low of 3207 articles thus far in 2019. These articles were published by both single and multiple authors. Dentistry is an area many researchers across the globe are contributing to—a total of 81 countries published articles on this topic. The leading countries in terms of production are the USA, England, Germany, Denmark and India. The most-common keywords used in the articles researched were “humans”, “methods”, “adult”, “female”, “middle-aged” and “physiology”.

## Figures and Tables

**Table 1:** Yearly percentage of the articles, 2009 to 2019.

Year	Articles	Percentage (%)
2009	9325	8.9
2010	10,328	9.8
2011	10,866	10.4
2012	10,619	10.1
2013	10,986	10.5
2014	11,059	10.5
2015	10,486	10
2016	10,465	10
2017	9989	9.5
2018	7645	7.3
2019	3207	3.1

**Table 2:** Global Dental Publications, 2009–2019.

Type of Publication	Frequency	Percentage %	Mean ± Standard Deviation	*p*-Value	Mean ACR *
All publications	104,975	100	9543.18 ± 2315.99	>0.001	−7.36%
Journal article	63,600	60.58	5354.18 ± 1843.80	>0.001	4.17%
Comparative study	16,858	16.05	1531.55 ± 781.52	>0.001	−17.56%
Case report	11,334	10.8	1035.36 ± 336.37	>0.001	−10.89%
Editorial	2654	2.53	237.73 ± 79.46	>0.001	−6.11%
Letter	2393	2.28	204.45 ± 99.49	>0.001	11.47%
Evaluation studies	1794	1.71	162.18 ± 62.15	>0.001	−11.01%
Clinical Trial	1114	1.06	104.18 ± 31.80	>0.001	−2.88%
News	521	0.5	49.09 ± 30.77	>0.001	−8.28%
Historical article	494	0.47	51.27 ± 32.29	>0.001	−15.97%
Biography	465	0.44	41.45 ± 22.92	>0.001	−11.73%
Controlled clinical trial	291	0.28	25.27 ± 13.42	>0.001	−15.69%
Interview	280	0.27	24.64 ± 15.89	>0.001	−2.72%
Others	3177	3.03	721.82 ± 1018.89	>0.001	31.61%

* ACR: Average Change Rate calculated (Current Year Total—Previous Year Total)/Previous Year Total.

**Table 3:** Impact factor metrics of the most relevant sources having JCR.

Journal Name	No. of Articles	Eigenfactor Score	CiteScore	5-year JIF
*Am J Orthod Dentofacial Orthop*	2707	0.012	3.4	2.405
*Br Dent J*	2497	0.003	1.2	1.524
*J Oral Maxillofac Surg*	2222	0.017	2.8	2.020
*J Endod*	2093	0.016	6.2	3.380
*J Prosthet Dent*	1935	0.005	4.4	2.727
*Int J Oral Maxillofac Implants*	1610	0.010	3.4	2.987
*J Craniofac Surg*	1543	0.012	-	1.050
*Angle Orthod*	1321	0.007	3.3	2.022
*Clin Oral Investig*	1295	0.009	4.4	2.710
*J Am Dent Assoc*	1276	0.006	4.3	2.950
*Dent Mater*	1223	0.014	8.0	5.386
*J Dent*	1188	0.011	5.8	4.265
*Int J Oral Maxillofac Surg*	1174	0.010	3.7	2.987
*J Periodontol*	1158	0.013	5.2	3.614
*J Dent Educ*	964	0.002	2.1	1.371

Journal Citation Report 2019. Abbreviation: JIF = Journal impact factor, Source for impact factor https://www.jcr.clarivate.com.

**Table 4:** Most relevant non-JCR sources.

Source	No. of Articles
*J Oral Maxillofac Res*	1824
*Dent Today*	1143
*J Contemp Dent Pract*	1135
*Int J Prosthodont*	1086
*Indian J Dent Res*	987

**Table 5:** The table further shows the same statistics for nine subsequent countries in our study.

Country	Total Published Articles for Each Country	Publications with Authors from Single Country	Publications with Authors from Multiple Countries	Total Citations for Each Country	Average Article Citations for Each Country
USA	10,281	9483	798	36,082	3.51
Brazil	9021	8086	935	23,029	2.55
China	5065	4366	699	9656	1.91
Germany	3943	3434	509	12,092	3.07
India	3808	3654	154	7770	2.04
Japan	3642	3386	256	8779	2.41
Turkey	3503	3364	139	8765	2.5
Italy	3475	3007	468	11,306	3.25
Korea	2096	1783	313	6081	2.9
Spain	1760	1520	240	5209	2.96

**Table 6:** Most Productive Authors’ h-indices and Total Citations.

Author	h-Index	Total Citations (TC)	Affiliation
Wang, Y	14	1301	Sichuan University
Zhang, Y	16	1154	New York University College of Dentistry
Liu, Y	13	891	Peking University School of Stomatology
Wang, H. L.	11	756	University of Michigan School of Dentistry
Wang, X	13	852	Capital Medical University
Li, J	14	1729	Yonsei University Wonju College of Medicine
Li, Y	12	614	Loma Linda University School of Dentistry
Piattelli, A	10	750	University of Chieti-Pescara
Lang, N. P.	17	1083	The University of Hong Kong
Tagami, J	10	504	Tokyo Medical and Dental University

**Table 7:** Top ten manuscripts with most citations.

Paper	Total Citations (TC)
EPSTEIN, L.J, 2009, J CLIN SLEEP MED	475
EKE, P.I, 2012, J. DENT. RES.	359
SHEIHAM, A, 2009, COMMUNITY DENT HEALTH	250
HABIB, G, 2015, EUR, HEART J.	246
PARIROKH, M, 2010, J ENDOD-a	198
RUGGIERO, S.L, 2014, J.ORAL MAXILLOFAC. SURG.	195
SCHIFFMAN, E, 2014, J.ORAL FACIAL PAIN HEADACHE	192
VOLZKE, H, 2010, INT J EPIDEMIOL	191
FERRACANE, J.L, 2010, DENT MATER	189
COLOMBO, A.P, 2009, J.PERIODONTOL	186
